# The β-Lactamase Assay: Harnessing a FRET Biosensor to Analyse Viral Fusion Mechanisms

**DOI:** 10.3390/s16070950

**Published:** 2016-06-23

**Authors:** Daniel M. Jones, Sergi Padilla-Parra

**Affiliations:** Division of Structural Biology, University of Oxford, The Henry Wellcome Building for Genomic Medicine, Headington, Oxford, OX3 7BN, UK

**Keywords:** β-lactamase, BlaM, CCF2-AM, FRET, fusion, kinetics, virus

## Abstract

The β-lactamase (BlaM) assay was first revealed in 1998 and was demonstrated to be a robust Förster resonance energy transfer (FRET)-based reporter system that was compatible with a range of commonly-used cell lines. Today, the BlaM assay is available commercially as a kit and can be utilised readily and inexpensively for an array of experimental procedures that require a fluorescence-based readout. One frequent application of the BlaM assay is the measurement of viral fusion—the moment at which the genetic material harboured within virus particles is released into the cytosol following successful entry. The flexibility of the system permits evaluation of not only total fusion levels, but also the kinetics of fusion. However, significant variation exists in the scientific literature regarding the methodology by which the assay is applied to viral fusion analysis, making comparison between results difficult. In this review we draw attention to the disparity of these methodologies and examine the advantages and disadvantages of each approach. Successful strategies shown to render viruses compatible with BlaM-based analyses are also discussed.

## 1. Introduction

**Figure 1:**
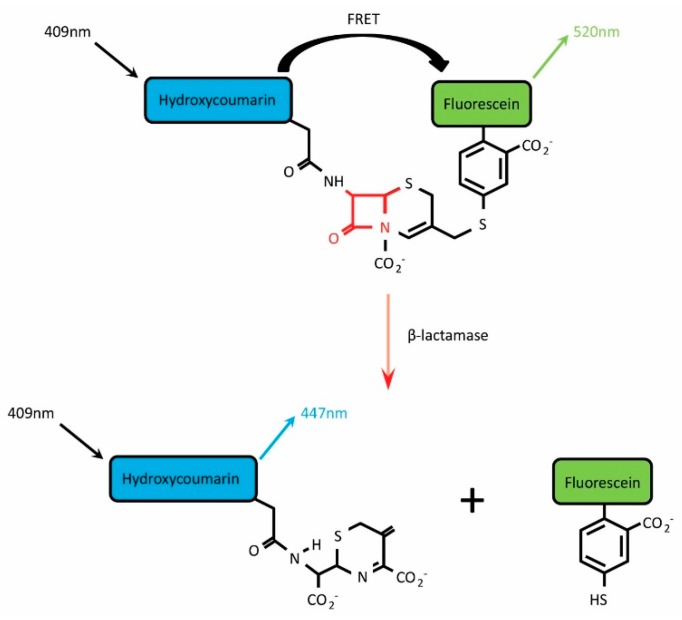
CCF2-AM is composed of a Hydroxycoumarin donor conjugated to a Fluorescein acceptor via a β-lactam ring. In the absence of β-lactamase, excitation at 409 nm promotes Förster resonance energy transfer (FRET) between the fluorescent donor and acceptor molecules, resulting in emission at 520 nm. Cleavage of the β-lactam ring by β-lactamase separates the two molecules, disrupting FRET and producing a fluorescence shift from 520 nm to 447 nm.

The β-lactamase (BlaM) assay is a commercially available system that relies on the action of β-lactamase on a Förster resonance energy transfer (FRET)-based substrate termed CCF2-AM (or the alternative substrate CCF4-AM). β-lactamases represent a family of bacterially-derived enzymes that cleave the β-lactam ring structure found within certain families of antibiotic including cephalosporins and penicillins, thereby deactivating them [1]. Since this enzymatic function promotes the development of bacterial antibiotic resistance, β-lactamases have been the subject of thorough research efforts that have in turn provided a plethora of information regarding their structures, enzymatic mechanisms and substrate specificities. Drawing upon this knowledge, in 1998 Zlokarnik and colleagues focussed on the TEM-1 β-lactamase from *Escherichia coli* (product of the ampicillin resistance gene, *AmpR*) and designed a suitable reporter substrate that would undergo rapid and efficient cleavage when the enzyme was present, and report β-lactamase-induced cleavage with a substantial shift in the wavelength of fluorescence emission [2]. The resulting substrate was termed CCF2-AM and consists of two fluorophores, hydroxycoumarin and fluorescein, attached to the 7’ and 3’ positions, respectively, of a cephalosporin β-lactam ring (Figure 1). In the absence of β-lactamase, excitation of hydroxycoumarin (acting as donor molecule) at 409 nm results in energy transfer by FRET to fluorescein (acceptor molecule), causing it to emit light in the green region of the spectrum (emission peak at 520 nm). However, exposure to β-lactamase promotes hydrolysis of the CCF2-AM β-lactam ring and separates the 3-fluorescein from the remainder of the substrate. In this scenario, FRET is disrupted and excitation of hydroxycoumarin leads to direct emission at 447 nm [2].

The BlaM assay offers a widely applicable system where a need exists for fluorescence-based readout from a sensitive FRET biosensor. In this review, we will discuss how the BlaM reporter can be used specifically to measure viral fusion—the prerequisite step of infection where the viral and cellular membranes coalesce to provoke intracellular release of the viral genetic material. 

## 2. Producing BlaM-Compatible Viruses

There are two essential requirements that must be fulfilled in order to measure viral fusion using the BlaM assay, namely (i) the target cell must be loaded with the CCF2-AM reporter substrate and (ii) the β-lactamase enzyme must be successfully incorporated within the viral particle of interest. The lipophilic nature of CCF2-AM makes it readily and easily deliverable into a wide range of cell lines and primary cells (some examples are listed in Table 1). A more difficult task, however, is to manipulate virus particles into packaging the 29 kDa β-lactamase protein. Viruses differ substantially in their ability to tolerate the incorporation of foreign proteins into assembling virions and while some may behave no different from their wild-type counterparts following modification, others may become partially or completely compromised in their infectivity. 

**Figure 2:**
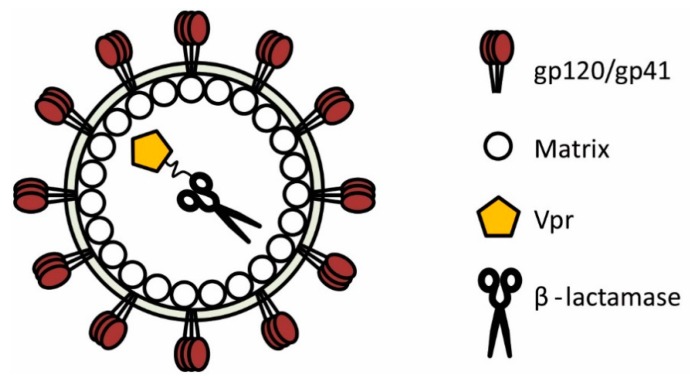
A representation of a HIV-1 particle is shown. β-lactamase can be packaged into nascent HIV-1 virions by fusing it to the accessory protein Vpr. For purposes of clarity, several HIV-1 proteins are not depicted.

Human immunodeficiency virus type-1 (HIV-1) displays a remarkably plastic assembly process and assembled virions harbour a multitude of accessory proteins, some of which are amenable to significant modification [3]. With this in mind, researchers utilised HIV-1 to produce a landmark study demonstrating that β-lactamase could indeed be successfully recruited into assembling virions, thereby permitting the study of HIV-1 fusion in a variety of relevant cells [4]. This key to this achievement was to fuse β-lactamase to the N-terminus of the HIV-1 viral protein R (Vpr, Figure 2), a multifunctional accessory protein that is recruited into virions in relatively large numbers (100–300 or more copies [5,6,7]) through interaction with the p6 domain of Gag [8,9,10]. The fusion of β-lactamase to the Vpr protein (producing BlaM-Vpr) represented a highly advantageous scenario; not only was the enzymatic activity of β-lactamase unaffected by its conjugation to Vpr, but the Vpr protein itself remained functional [4]. Consequently, incorporation of BlaM-Vpr into assembling particles represents a robust method for ensuring the complete HIV-1 life cycle remains intact whilst rendering the virus permissive to sensitive fusion measurements using the BlaM assay. The success behind this approach has been replicated by researchers worldwide and to date the BlaM assay has been applied to HIV-1 more than any other virus, providing a wealth of information with respect to HIV-1 entry and fusion.

HIV-1 is somewhat unique regarding the sheer number of proteins that are physically encapsulated within its infectious particles and by comparison, other viruses typically package fewer proteins that can be less amenable to modification than Vpr. For viruses where β-lactamase is difficult or even impossible to enclose, an alternative strategy involves the exploitation of another useful HIV-1 property—the virus’s ability to create virus-like particles (VLPs), also known as pseudoparticles. Whereas many viruses rely on the combined contribution of several viral proteins to drive virion assembly, expression of the HIV-1 Gag protein alone is sufficient for the production of VLPs that are morphologically similar to immature wild-type HIV-1 particles [36,37]. Because the HIV-1 envelope proteins gp120 and gp41 are unimportant for this process, they can be substituted for those from other viruses. Thus, HIV-1 VLPs remain composed of a Gag protein core (preserving the ability to bind and package BlaM-Vpr) surrounded by a lipid envelope, yet will exhibit the entry and fusion characteristics inherent to the substituted envelope proteins. Although this system is not compatible with all foreign envelopes, HIV-1 VLPs expressing envelope proteins from vesicular stomatitis virus (VSV [20,22,35,38]), influenza A virus (IAV [11,24]), Ebola virus (EBOV [11,22]) and Lassa virus (LASV [11]) have all been successfully generated and probed using the BlaM assay. 

More recently, several non-HIV-1-based VLP systems capable of packaging β-lactamase have been reported. For example, expression of the two envelope proteins alone from IAV—hemagglutinin (HA) and neuraminidase (NA)—is sufficient to drive production of VLPs that are morphologically similar to wild-type influenza viruses [39]. Whilst the IAV matrix protein, M1 (which forms the viral capsid), is dispensable for VLP production, it will be packaged within VLPs if present [39]. Accordingly, an M1-BlaM fusion protein can be generated that will be drawn into VLPs through the interaction between M1 and HA, creating influenza VLPs that should more closely mimic bona fide IAV particles compared to HA- and NA-expressing HIV-1 VLPs [25]. *Filoviridae* family members Marburg virus (MARV) and EBOV can produce VLPs compatible with BlaM analysis by expressing β-lactamase-tagged versions of their matrix proteins, VP40 (producing BlaM-VP40), in combination with their respective envelopes [25,40]. Similarly, the matrix proteins of Nipah (NiV) and Hendra (HeV) virus can be fused to BlaM and will produce entry-capable VLPs when expressed alongside their receptor binding (G) and fusion (F) proteins [12,13]. For many of these viruses, working with VLPs as opposed to genuine viruses has an added advantage-EBOV, MARV, NiV and HeV are all highly dangerous pathogens that can only be worked with under biosafety level (BSL) 4 conditions, making any type of investigative biological assay cumbersome. Because VLPs do not replicate their genomes or produce infectious progeny, BlaM-based entry studies can be conducted without the need for such stringent safety requirements. 

**Figure 3:**
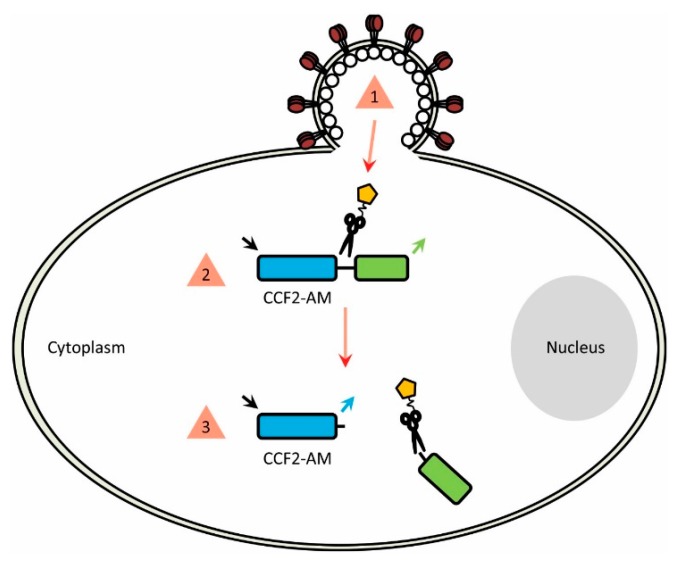
(1) Fusion between the virus particle and target cell (either at the cell membrane or from within endosomes) liberates the encapsulated β-lactamase (2) The enzyme is then able to access the cytoplasmic CCF2-AM FRET substrate (3) CCF2-AM cleavage occurs and the fluorescence profile is altered, indicating fusion has occurred.

Once entry-competent viruses/VLPs packaging β-lactamase have been successfully produced, they can be incubated with CCF2-AM-loaded cells. Such a strategy ensures that β-lactamase will be released from the virion at the moment viral fusion occurs, allowing it to access and cleave the cytoplasmic CCF2-AM and produce a change in emission profile that can then be recorded (Figure 3).

## 3. Choosing a BlaM-Based Viral Entry Assay

Before dissecting how BlaM-based viral fusion assays are conducted, a few fundamental aspects of virus entry and fusion should first be outlined:

- The ratio of infectious virus particles to cells is referred to as the multiplicity of infection (MOI).
- At 4 °C, virus particles are able to bind to target cells but are not able to enter them [41]. A 4 °C incubation step is therefore often used to prime virions on the surface of the cell, meaning any unbound particles can be washed away.
- Viral fusion is initiated by a temperature shift from 4 °C to 37 °C [42].
- Spinoculation refers to the practice of centrifuging cells in the presence of virus particles during the 4 °C priming step. Several reports agree that this technique boosts viral infectivity compared to when virus-cell interaction occurs under normal gravitational conditions [43,44,45].

BlaM assays can be broadly divided into two categories; fusion endpoint and fusion kinetic assays. For a fusion endpoint assay, a single measurement is made to determine the total amount of fusion produced by a virus after a specified period of time. Conversely, a fusion kinetic assay sees the acquirement of multiple measurements over time in order to provide information on the rate of fusion. Fusion kinetic assays can provide more informative data than fusion endpoint assays where required. For instance, viruses A and B may produce the same total fusion levels by 4 h, and therefore look identical in a fusion endpoint assay. However, virus A might reach its fusion plateau with twice the rapidity of virus B, an important property that would only be revealed by a fusion kinetic assay. A second example might concern the screening of potential fusion inhibitors; an endpoint assay might suggest that a candidate viral fusion inhibitor has no effect on viral fusion, yet it may become evident during a kinetic assay that the compound slows fusion rather than halting it completely. For rapid determination of IC_50_ values (the concentration of drug required to reduce virus fusion by half) for any given fusion inhibitor however, titration of the compound coupled with an endpoint assay is sufficient [46]. 

**Figure 4:**
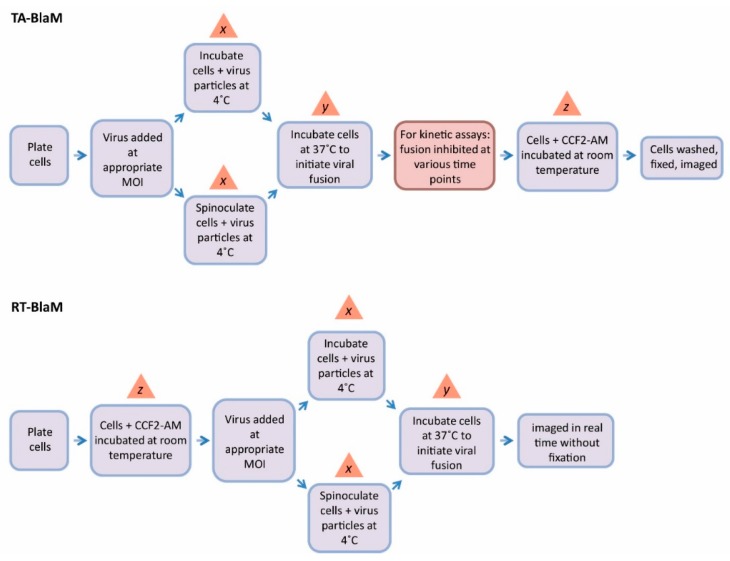
Methodology pipelines for the TA-BlaM and RT-BlaM assays. Virus particles are allowed to fuse before the addition of CCF2-AM in the TA-BlaM assay, and cells are typically fixed before analysis. The red box in the TA-BlaM flow diagram applies to fusion kinetic assays and is omitted under fusion endpoint assay conditions. In a RT-BlaM assay, target cells are loaded with CCF2-AM before being exposed to virus, meaning fusion can be monitored in live cells. The timing used for several steps (*x,y* and *z*) vary and are discussed in more detail in Section 4 of the main text. MOI = multiplicity of infection.

For all fusion endpoint assays and the majority of fusion kinetic assays that are referenced in this review, the majority utilise a methodology pipeline that, for simplicity, will be referred to here as the ‘time-of-addition BlaM’ (TA-BlaM) assay. For fusion kinetic assays however, a seemingly less popular yet equally valid technique is also reported in the literature–here, we call this the ‘real-time BlaM’ (RT-BlaM) assay (see Figure 4 for the methodology underlying each).

**Figure 5:**
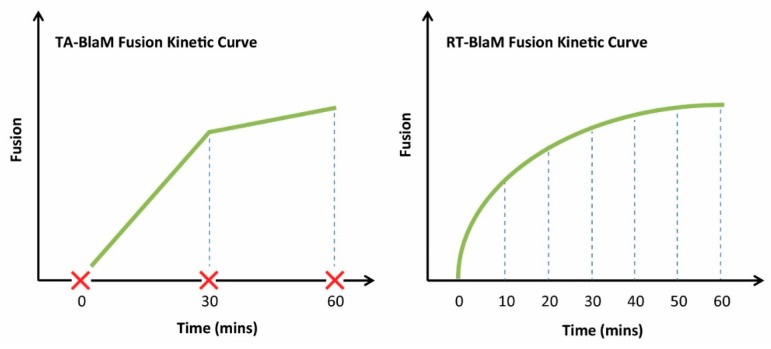
To obtain kinetic measurements of fusion using a TA-BlaM assay (**Left**), fusion must be stopped at various time points using a known fusion inhibitor (red cross). The level of fusion that occurred up to the time of inhibition can then be quantified. In a RT-BlaM assay (**Right**), fusion can be measured in real time in the absence of fusion inhibitors.

The TA-BlaM assay is by far the most commonly used method employed to measure viral fusion and is compatible with both endpoint and kinetic assays. The most important point to consider when utilising a TA-BlaM approach is that viral entry and fusion occurs prior to addition of the CCF2-AM reporter substrate and not after. Such an approach seems illogical, especially where fusion kinetic assays are concerned—how can a kinetic curve be elucidated if fusion is permitted to proceed before the CCF2-AM substrate is even present? To do this, inhibitory concentrations of known fusion-blocking compounds must be added at specific time points (hence the term ‘time-of-addition’) to distinct samples of cells/virus. For instance, a relatively simple fusion kinetic curve could be generated by incubating three samples of cells/virus and blocking them 5, 30 and 60 min after fusion is initiated (Figure 5, left panel). This would mean that when all cells are subsequently loaded with CCF2-AM, the resulting level of fusion would be indicative of what took place up to the point at which the inhibitor was added, and the three datasets can then be combined to produce a curve revealing viral fusion levels over time. By contrast, the RT-BlaM assay represents a more streamlined approach for measuring virus fusion kinetics. Here, target cells are first loaded with the CCF2-AM FRET substrate and then exposed to virus particles. This means upon temperature shift to 37 °C, cleavage of CCF2-AM and the resultant colour change from green to blue can be visualised in real time, all in a single sample of cells/virus and without the need for fusion inhibitor addition. This typically permits the recording of more datasets and produces a more refined kinetic curve (Figure 5, right panel).

It is abundantly clear that the TA-BlaM method represents the most popular virus fusion assay since very few reports describe using the RT-BlaM approach [12,31]. Why then is the RT-BlaM assay used so infrequently, especially when it is seemingly tailored to the analysis of fusion kinetics? One possible reason is that many researchers may have simply repeated what is essentially a validated method in the TA-BlaM assay, since this was the original method described in the report detailing the use of the BlaM assay to measure HIV-1 fusion [4]. Another potential reason concerns the instrument used to collect the data; the fact that the BlaM assay produces fluorescence-based readout means that measurements can be acquired through a variety of means including microscopy [15,19,23,35], flow cytometry [4,17,22,24,26,38] or plate reader-based analyses [11,12,14,16,18,30]. Since live cell analysis is central to the success of the RT-BlaM assay, the chosen platform must be capable of providing a temperature of 37 °C (and preferably a source of 5% CO_2_ for the sake of cell viability) so that fusion and CCF2-AM cleavage can occur simultaneously with data acquirement. Such resources may not be available to all researchers, or it possible that some prefer the fixed-cell approach inherent to the TA-BlaM assay, where data can be collected over a longer period of time and concerns for cell viability and CCF2-AM loss (detailed in Section 4) are largely avoided. It is interesting to note that the RT-BlaM method has appeared relatively recently and it’s therefore entirely possible that the technique will become more popular once its merits are fully realised and when technology supporting live cell fluorescence analyses becomes more widespread. A summary of the TA- and RT-BlaM assays alongside the strengths and drawbacks of each are presented in Table 2.

## 4. BlaM Assay Intricacies and Discrepancies

Regardless of the method chosen, for any BlaM assay to be valid it is imperative that a significant difference in fusion levels exists between the virus being tested and an appropriate fusion-negative control (e.g., media only, or entry-incapable particles that lack envelope proteins). With this goal in mind, several assay parameters can be tweaked in order to maximise assay sensitivity. Using a higher MOI is one simple variable and can provoke increased fusion compared to when lower concentrations of virus are used [35]. One area that offers a large degree of fine-tuning concerns the incubation times allocated to virus binding, entry and CCF2-AM cleavage (steps *x, y* and *z*, respectively, in Figure 4) and perhaps unsurprisingly, substantial discrepancies in the timings applied can be found in the literature. Firstly, the 4 °C incubation step designed to prime virus particles on the surface of target cells (step *x*) ranges from as little as 30 min at atmospheric gravity [31] to 2 h of spinoculation at 2000 xg [13], with almost every variation in between. Similarly, the time permitted for virus entry/fusion (step *y*) extends from 45 min in some studies [15] to 6 h in others [16]. Perhaps the greatest difference can be seen in the CCF2-AM incubation step (step *z*), which can be as little as 1 h [24] or as long as 18 h [13]. Overall, these variables combined with the presence or absence of spinoculation offer great scope for achieving a suitable dynamic range between tests and controls.

Another technique to aid BlaM analysis involves the use of probenecid, a non-specific inhibitor of anion transport that can aid the retention of CCF2-AM within target cells. Following intracellular delivery, esterified CCF2-AM is cleaved by endogenous esterases and rapidly converted into the negatively charged form, which should be more readily retained in the cytoplasm [2]. Despite this, the green substrate and blue product are both removed from the cell by nonspecific anion transporters and furthermore, the removal of each occurs at different rates. For instance, the original BlaM report cited a 50% loss of the green substrate from Jurkat cells by 2.5 h, whereas 50% of the blue cleavage product was removed only 1.5 h after loading [2]. Conversely, the blue product was retained more effectively than the green substrate in CHO cells; no matter the pattern, all of these effects could be diminished through the use of probenecid [2]. It should be noted that CCF2-AM expulsion increases at 37 °C and can therefore be minimised by performing the substrate loading (step *z* in Figure 4) at room temperature during TA-BlaM assays. Regardless, many researchers choose to use probenecid in combination with room temperature incubation in order to maximise CCF2-AM retention [4,17,22,29]. In a RT-BlaM assay, however, conducting step *z* at anything less than 37 °C for 1–2 h is unavoidable given that CCF2-AM incubation and virus fusion must occur simultaneously, making the use of probenecid almost mandatory. 

One other option for improving BlaM readout is to enhance the action of the intra-viral β-lactamase enzyme itself. One study highlights how introducing a single point mutation (Y105W) into the β-lactamase sequence is sufficient to enhance the sensitivity of the assay by approximately 2-fold [13]. Alternatively, a codon optimization approach can ensure that the protein is expressed to optimal efficiency in mammalian cells [12,13]. Finally, it is also possible to swap CCF2-AM with other customised substrates where the hydroxycoumarin and fluorescein fluorophores are exchanged for other FRET substrates. Instead of offering a change in colour for example, a biosensor designed to be better suited for imaging tuberculosis in live mice resides in a dark state until β-lactamase-induced cleavage provokes fluorescence emission in the near-infrared region of the spectrum [49]. Another β-lactamase FRET substrate features Cy5 paired with a quantum dot (QD)—nanosize semiconductor crystals offering improved fluorescence brightness and stability compared to conventional fluorophores [50]. To date however, none of these modified β-lactamase-compatible FRET biosensors have been applied to the investigation of viruses specifically. It will therefore be interesting to see in the future whether any of these improved β-lactamase substrates can further enhance the detection of viral fusion using the BlaM assay. 

## 5. BlaM and Other Methods of Viral Fusion Analysis

Although the BlaM assay has been adopted widely throughout the research community, it is important to note that it is not the only means of measuring viral fusion. What other methods exist and how do they compare to the BlaM assay?

Single virus tracking (SVT) represents what might possibly be the gold standard for measuring the fusion event between virus particles and cells (see [51] for a detailed review) and is compatible with live cells as well as artificial membrane models [52]. Here, the virus of interest is itself fluorescently labelled; typically through the incorporation of a lipophilic tracer dye (Dil, DiO, DiD, etc.) that embeds within the viral membrane or amine-reactive dyes [53], and/or by manipulating the virus particle to package a fluorescent protein such as GFP or mCherry in a manner analogous to that used for β-lactamase inclusion [15,54]. Introducing a fluorescent protein into the virion is subject to all the same challenges previously described for β-lactamase, whereas chemical compounds should readily label any viruses present in the solution. However, lipophilic and amine-reactive dyes will also non-specifically associate with all lipid- or amine-containing species found within viral supernatants, meaning viral purification processes may be necessary to reduce background fluorescence to acceptable levels [53,55]. For precise exploration of fusion properties, labels (i.e., multiple dyes or dyes and proteins) can be combined to generate dual-labelled particles that can be tracked and analysed in unparalleled detail. For example, the pH-dependent fusion of avian sarcoma and leukosis virus (ASLV) from within endosomes has been closely followed by tracking particles featuring GFP-expressing capsids (green) and DiD-labelled envelopes (red) [15]. Dual-labelled (yellow) particles can be followed in real-time; fusion has occurred when a shift from yellow to red is observed, a scenario that arises from the virion contents being released into the cytoplasm (resulting in loss of the GFP signal) whilst the DiD undergoes limited dilution due to its redistribution into the endosomal membrane [15]. If a double-labelling strategy is unavailable, fusion can also be pinpointed through the phenomenon of fluorescence dequenching—a spike in fluorescence intensity that occurs when the viral and cellular membranes merge, as demonstrated by studies on DiD-labelled IAV [53,56] and Dengue virus (DENV) [57]. The most obvious advantage of SVT is that the fusion event is observed and recorded at the very moment it occurs, whereas in the BlaM assay a delay between fusion and detection is expected since the CCF2-AM substrate must first be accessed and cleaved in detectable quantities, a process that can only occur once β-lactamase has been released following the fusion event. Another advantage of SVT is that information on fusion is derived from the viral particle itself and not from the target cell, as is the case with BlaM. The cell-based readout inherent to the BlaM assay makes it difficult to discern whether successful substrate cleavage was driven by single or multiple fusing particles, whereas information yielded by SVT analyses are on a per-virion basis. In other words, the BlaM assay reports a large number of fusion events at once, and therefore cannot provide the resolution that SVT is capable of. SVT however does have significant caveats; microscopy equipment featuring live-cell functionalities and supporting single molecule sensitivity with high spatial resolution is essential for the detection and tracking of single viruses, making the technique inaccessible to many researchers. Although a finer level of detail can be resolved with SVT, gathering individual virus fusion events in sufficient enough quantities to make a statistically sound conclusion is especially time-consuming, much more so than using the BlaM assay.

In addition to virus-cell fusion assays, such as BlaM and SVT, so called virus-free or cell-cell fusion assays have proven to be a popular surrogate system in the fusion field. In these assays, the approach typically consists of mixing target cells (expressing the appropriate viral receptor) with effector cells (a different type of cell manipulated to express the viral envelope protein(s) of interest, usually alongside an enzymatic or fluorescent reporter) before using the reporter expression in the target cells as a marker for successful membrane fusion [58,59,60]. The transfer of a fluorescent protein or dye between cells is preferable to an enzymatic reporter because this event proceeds immediately following fusion. Although broad conclusions regarding the functionality of viral envelope proteins and their role in fusion might be made, it is unlikely that cell-cell fusion assays provide an exact picture of reality. For example, some viral envelope proteins might orientate slightly differently on or across a cellular membrane compared to how they would sit on a viral envelope. Moreover, the surface area of a cell is obviously much greater than that of a virus—what implications might these factors have for protein-receptor interactions, and consequently, for membrane fusion? Regardless, these virus-free assays have provided valuable data and have been particularly useful where safety issues are of concern as with EBOV [61,62], or where virus might be difficult to propagate in cell culture—Epstein-Barr virus (EBV) for instance [63,64].

It should be noted that assays detecting viral components (such as p24 in HIV-1) or virally-encoded reporter genes (such as luciferase) first rely on the successful replication of the virus being examined and are therefore regarded as infectivity assays rather than fusion assays. Accordingly, such techniques will not be discussed further in this review. A comparison between these different methods of viral fusion analysis is depicted in Table 3. 

## 6. Conclusions and Future Directions

The BlaM assay represents an attractive FRET-based assay for researchers investigating the characteristics and timing of the fusion event occurring between virus particles and their target cells. The kit is commercially available, seemingly compatible with all types of cell, sensitive and produces a robust output that can be interpreted by any platform provided it features the correct excitation and emission filters. The assay has been integral to a multitude of fusion-centric discoveries including the importance of endocytosis and endosomes during HIV-1 entry in addition to the role of interferon-induced transmembrane (IFITM) proteins in inhibiting HIV-1 and IAV fusion. The BlaM assay has also been demonstrated to be particularly well suited to high-throughput screening (HTS) assays designed to identify potential fusion inhibitors, as demonstrated for HIV-1 [21] and EBOV [40]. While it is beyond the scope of this review to detail all relevant findings, it is clear that the BlaM assay has proved to be an invaluable tool for investigatory virology and it is highly probable that its use will contribute to further important discoveries in the future. 

For those using the BlaM system to research virus fusion events, finding a suitable method for incorporating β-lactamase into the particle of interest likely represents one of the more difficult challenges to overcome. Although VLP-based studies provide the bulk of success stories, other novel and imaginative methods should be sought with the aim of producing BlaM-compatible viruses that more closely represent bona fide virions during all stages of infection. Such a strategy would allow researchers to extrapolate their findings beyond fusion and toward genome replication and virus assembly/egress, and would prove particularly useful for the study of viral fusion inhibitors where any additional inhibitory effects on these latter live cycle stages could be assessed. 

Like any assay, The BlaM assay offers enormous flexibility to the user and this is reflected in the diversity of methods employed in the literature with regard to incubation times, probenecid use, β-lactamase enhancement and spinoculation. However, the application of such vast methodological differences makes comparing disparate fusion studies difficult, even when the same virus is the subject of investigation and caution is therefore warranted if this is to be attempted. One final point of importance is that in addition to improving the virus priming, spinoculation may well perturb the biology of the target cell itself [65]. Thus, it cannot be excluded that any insight into viral fusion gained from assays conducted in this fashion may be unrepresentative of those that would be seen naturally. Accordingly, investigators may wish to use spinoculation as a last resort when all other variables have failed in producing a working assay.

## Figures and Tables

**Table 1:** Examples of cell lines and primary cells (denoted by *) successfully loaded with CCF2-AM and subsequently used for viral fusion analyses.

Cell Type	Literature Example
A549	[11]
CD4+ T cells *	[4,5,6,7]
CEMss	[6,7,8]
CHO	[2,11,12,13]
COS-7	[2]
CV-1	[2,11,14,15]
HEK 293T	[2,16]
HeLa	[2,4,17,18,19,20,21,22]
HMVEC*	[13]
Jurkat	[2,4,23]
MDCK	[11,24,25]
MT-4	[18,26]
PBMC *	[4,16,22,23,24]
PM-1	[23]
SupT1 T cells	[4,17,27,28,29]
TZM-bl	[15,20,21,23,30,31,32,33,34,35]
U87 CD4 + CCR5 +	[23,31]

**Table 2:** A comparison of the features, advantages and disadvantages associated with the TA- and RT-BlaM assays.

	Main Feature	Literature Example	Advantages	Disadvantages
**TA-BlaM**	Virus fusion precedes CCF2-AM loading	[4,11,13,14,15,16,17,18,19,20,21,22,23,24,25,26,27,28,29,30,32,33,34,35,38,47,48]	Best suited for fusion endpoint assays. Kinetic assays still possible Extensively validated Compatible with flow cytometry, microscopy and plate readers	One sample per time point required for kinetic analyses: greater reagent usage Kinetic measurements require the use of fusion inhibitors
**RT-BlaM**	CCF2-AM loading precedes virus fusion	[12,31]	Best suited to fusion kinetic assays Kinetic analyses can be conducted on a single sample Use of fusion inhibitors is optional Easier to acquire more measurements: more refined curves can be generated	Data collection platform should ideally support live cell analyses (37 °C, 5% CO_2_) Probenecid is likely required to limit CCF2-AM loss during 37 °C virus fusion step Data must be acquired in real time: No room for error

**Table 3:** A comparison of the benefits and drawbacks of several popular methods used to measure viral fusion.

	Advantages	Disadvantages
**BlaM Assay**	Data acquirement not limited to microscopy—plate readers and flow cytometers work efficiently Flexible protocols can be applied	Does not provide single virus precision Fusion detection may be slightly delayed
**SVT**	Fusion measured with single virus precision Instantaneous observation of fusion event	Extremely Time consuming and cumbersome Requires high-end microscopy equipment and SVT expertise Multiple events required to draw firm conclusions
**Cell-cell/Virus-free Assay**	Virus production and characterisation not required Relatively straightforward protocol compared to BlaM and SVT	Cells unlikely to accurately mimic virus particles—caution with data interpretation required

## Abbreviations

The following abbreviations are used in this manuscript:

ASLV	Avian sarcoma and leukosis virus
FRET	Förster resonance energy transfer
EBOV	Ebola virus
EBV	Epstein-Barr virus
GFP	Green fluorescent protein
HeV	Hendra virus
HIV-1	Human immunodeficiency virus-1
HTS	High-throughput screening
IAV	Influenza A virus
IFITM	Interferon-induced transmembrane protein
LASV	Lassa virus
MARV	Marburg virus
MOI	Multiplicity of infection
NiV	Nipah virus
QD	Quantum dot
RT-BlaM	Real-time BlaM assay
TA-BlaM	Time-of-addition BlaM assay
VLP	Virus-like particle
Vpr	Viral protein R
VSV	Vesicular stomatitis virus
